# Measuring the Timing of the Bilingual Advantage

**DOI:** 10.3389/fpsyg.2018.01983

**Published:** 2018-10-16

**Authors:** Sara Incera

**Affiliations:** Multilingual Laboratory, Department of Psychology, Eastern Kentucky University, Richmond, KY, United States

**Keywords:** bilingualism, timing, eye tracking, mouse tracking, event-related potentials

## Abstract

Empirical evidence has supported the idea that the bilingual advantage is a question of nuanced differences between bilinguals and monolinguals. In this article, I review findings from studies using eye tracking, mouse tracking, and event-related potentials (ERPs) which are particularly suited to measure time. Understanding the timing of the processes underlying executive function is crucial in evaluating the intricacies of the bilingual mind. Furthermore, I provide recommendations on how to best use these timing techniques to compare bilinguals and monolinguals. Temporal differences can characterize ongoing discussions of the bilingual advantage and help explain conflicting findings. Methodological and analytical innovations to better investigate the timing of the cognitive processes at play will inform a wide range of areas in cognitive science.

## Introduction

More than half of the world’s population is bilingual (Grosjean, 2010). Studying the cognitive processes (e.g., executive function, conflict monitoring) underlying the bilingual mind is an important topic. The bilingual advantage refers to the idea that being bilingual is linked to cognitive benefits (for a review see Bialystok, 2017). However, there are researchers that have challenged this idea (Paap and Greenberg, 2013; de Bruin et al., 2015; Paap, 2015). In light of the debate over the bilingual advantage, there is a need for a more nuanced explanation of the consequences of bilingualism. It is crucial to take into account information regarding who the bilinguals and monolinguals are (Luk and Biaylstok, 2013), the types of experimental tasks implemented, the particular cognitive resources that may be critical to bilingualism (Takahesu Tabori et al., 2018), and the contexts in which bilinguals learned and normally use their languages (Green and Abutalebi, 2013). In addition to all of these variables, and possibly interacting with many of them, researchers need to consider the timing of the cognitive processes underlying participants’ responses.

The focus of the present paper is the timing of the cognitive differences between bilinguals and monolinguals. By timing I refer to the first one second (1,000 ms) of participants’ responses. Even though an important endeavor for researchers is to investigate the bilingual advantage over years or decades (Filippi et al., 2018; Incera and McLennan, 2018a), a review of those studies is beyond the scope of the current article. In addition, practice effects (Green and Abutalebi, 2013) and stimulus onset asynchrony manipulations (Martín et al., 2010) are likely to influence the bilingual advantage. However, investigations that do not measure participants’ responses as they unfold over time are beyond the focus of this review. When talking about timing in the present paper I am always referring to the unfolding of participants’ responses in milliseconds (ms). Using high temporal resolution techniques such as eye tracking, mouse tracking, and event-related brain potentials (ERPs), it is possible to analyze how each participant responds over time. Studying participants’ responses using time-sensitive techniques can guide the debate over the bilingual advantage by providing information about the timing of the cognitive processes at play.

Many researchers investigating the bilingual advantage have used experimental tasks in which the main outcome variable is reaction times (RTs). Typically, the dependent variable is the amount of time that participants take to complete a specific task, such as pressing a button after being exposed to visual or auditory stimuli. In this paper, I review studies that compare bilinguals and monolinguals using techniques that measure participants’ responses over time. Furthermore, I put forward methodological recommendations (see **Table 1**) that I believe will improve our understanding of the timing of the bilingual advantage. The goal of these suggestions is to better compare across studies using high temporal resolution measures. Triangulating across these techniques can generate new research questions and provide novel insights. A better understanding of the timing of the cognitive processes underlying executive function can help uncover nuanced differences between bilinguals and monolinguals.

**Table 1:** Summary of the recommendations covered in this article.

Design	Include a time-sensitive measure in your investigation
	Triangulate across measures and compare the results
	Collaborate with researchers who are proficient using other methods
	Control for variables that influence timing (language exposure/proficiency)
	Add a baseline condition before starting your experimental task

Analysis	Pre-register separate hypotheses for each time-sensitive measure

	Use factor analyses to combine different dependent variables
	Use factor analyses to combine different independent variables
	Decide whether you want to treat “time” as categorical or continuous
	Treat bilingualism as a continuous variable
	Focus on the interactions instead of the main effect of bilingualism
	Include trial presentation order as a control variable
	Report the raw (in ms) instead of the normalized mouse trajectories
	Report the time window of the effects instead of a single point in time
	Report stimulus-locked instead of response-locked ERP responses
	Create a section in the results to integrate across findings
	Develop statistical procedures to pinpoint the timing of the effects

Visualization	Create time figures that can be compared across measures

	Plot at least the first 1,000 ms of the responses (more if RTs are longer)
	Place time on the *x*-axis and the dependent variable on the *y*-axis
	Present different conditions as different lines
	Include lines for bilinguals and monolinguals within the same figure

## Eye Tracking

Eye tracking has been available as a research tool since the 1970s (Cooper, 1974), but eye tracking did not become a mainstream methodology in spoken language research until the 1990s (Tanenhaus et al., 1995; Tanenhaus and Spivey-Knowlton, 1996; Allopenna et al., 1998). Using the eye-tracking methodology, it is possible to measure “the probability of fixating a particular object as a function of time” (Tanenhaus and Spivey-Knowlton, 1996, p. 584). Researchers can analyze the total number of fixations on a specific area of the screen, or the proportion of fixations on areas of interest compared to control areas. Furthermore, it is typical to calculate the average number of fixations every 100 ms. Traditionally, eye-tracking figures include “time” on the *x*-axis and “proportion of fixations” on the *y*-axis (e.g., Allopenna et al., 1998). Most researchers report the first second (1,000 ms) of participants’ responses from target onset and represent the different conditions (e.g., fixations to each object) as separate lines. This way of representing the results has also been used when reporting mouse-tracking and ERP data, which makes this method a convenient way to compare results across methodologies.

Many of the eye-tracking studies with bilingual populations have focused on reading (Libben and Titone, 2009; Pivneva et al., 2014; Cop et al., 2017; Enkin et al., 2017; Indrarathne and Kormos, 2018) or auditory processing (Spivey and Marian, 1999; Marian and Spivey, 2003; Blumenfeld and Marian, 2007; Bartolotti and Marian, 2012; Ito et al., 2018). However, there are a few studies that have used eye tracking to test the bilingual advantage hypothesis for inhibitory control (Bialystok et al., 2006; Blumenfeld and Marian, 2011; Mercier et al., 2014; Blumenfeld et al., 2016).

Bialystok et al. (2006) measured executive control using an antisaccade task, an experimental paradigm in which response suppression is required to resist moving the eyes toward the briefly exposed target. These researchers performed two studies, each with 96 participants (24 monolingual young adults, 24 bilingual young adults, 24 monolingual older adults, and 24 bilingual older adults) recruited from their university research pool in Toronto. Bialystok et al. (2006) found no effects of aging or bilingualism when the eye-tracking task was presented in isolation (Study 1). However, they found a bilingual advantage that increased with age when the same visual display was coupled with keypress responses (Study 2). The authors explained this pattern by stating: “Saccadic eye movements are more rapid (150–350 ms) than button-pressing responses (350–650 ms) and are arguably more automatic and less amenable to higher level cognitive control” (Bialystok et al., 2006, p. 1352).

The fact that effects can emerge in button press but not in eye tracking is not limited to the bilingual advantage. For example, long-term repetition priming effects (responding to a word faster when you have heard that word in a previous block of trials) are very robust in button-press tasks but do not emerge in eye-tracking tasks. To my knowledge, no published study has reported long-term repetition priming in proportion of fixations over time. It follows that triangulation across methodologies is crucial toward gaining a better understanding of the nature of the effects found in such experiments. These apparently contradictory results are puzzling, but can be an opportunity to refine our theories. Using the same stimuli across different techniques researchers can explore what aspects of the task are driving the results.

Blumenfeld and Marian (2011) asked bilingual and monolingual participants to listen to words in English (their native language). For each trial participants had to identify the target word among four pictures, one of which was a similar-sounding within-language competitor (e.g., hamper/hammer). In the next trial the previously inhibited competitor picture became the target, a clever way to measure negative priming. In addition, participants responded to a version of the Stroop task in which they had to indicate the direction of an arrow. The arrow direction and arrow location could be congruent (leftward-facing arrow located on the left) or incongruent (leftward-facing arrow located on the right). These researchers reported a bilingual advantage in inhibitory control related to timing: “…bilinguals may return to a baseline activation state faster after inhibiting irrelevant information. In fact, the better bilinguals were at resolving Stroop interference, the less residual competitor inhibition they showed” (p. 11). Furthermore, they extended these findings to older adults: “…bilingual groups showed quicker target deactivation, reflecting more lifespan changes in activation for monolinguals than bilinguals” (Blumenfeld et al., 2016, p. 8). According to Blumenfeld and Marian (2011), the timing of inhibition (i.e., the time participants take to activate/deactivate a particular target) could be an important way in which bilinguals and monolinguals differ.

Mercier et al. (2014) monitored the eye movements of English monolinguals and French-English bilinguals while they listened to words in English. The non-target pictures included a within-language competitor, a between-language competitor, and a filler. Participants also responded to a battery of inhibitory control tasks. Mercier et al. (2014) reported a delayed onset of within-language competition for native French participants with low English exposure when compared to native English participants and to native French participants with high English exposure. According to these results, the timing of participant’s responses not only differs between bilinguals and monolinguals, timing differs between bilingual groups with unequal levels of language exposure. If you test bilingual participants in English, those with more experience using English will respond faster than those with less experience using English.

While these studies have made tentative conclusions about time and have supported the idea that the timing of bilinguals and monolinguals differs, the reporting of the results is heavily focused on overall responses. As it is typical in the literature, researchers report overall patterns across several hundreds of milliseconds. Furthermore, it is common to create a separate graph for each group (bilingual/monolingual) and then show the patterns for the different conditions (target/within-language competitor/cross-language competitor/filler). While this approach is very useful to understand lexical activation, it might fall short to understand bilingual effects. To better evaluate group differences researchers need to compare the unfolding patterns of bilinguals and monolinguals by plotting them within the same figure. This approach will make it possible to measure the time at which the responses of bilingual and monolingual participants diverge.

## Mouse Tracking

Mouse tracking is a tool that allows researchers to measure the unfolding of cognitive processes by recording participants’ computer mouse trajectories (Spivey et al., 2005). Since the landmark PNAS article, “Continuous attraction toward phonological competitors” (Spivey et al., 2005), researchers have applied the mouse-tracking paradigm to a wide range of cognitive tasks. In 2009, the open source software MouseTracker became publicly available (Freeman and Ambady, 2010), making the technology accessible to a larger number of researchers. More recently, Kieslich and Henninger (2017) developed Mousetrap, an OpenSesame plugin that facilitates the combination of mouse tracking with other techniques such as eye tracking. Within the open science framework, researchers are building online communities to increase the exchange of validated experimental tasks across teams, an approach that increases replicability. Furthermore, Mousetrap directly connects to the statistical programming language R, a feature designed to streamline data analysis (Kieslich and Henninger, 2017).

Mouse-tracking measures have been implemented with bilingual populations (Bartolotti and Marian, 2012; Incera and McLennan, 2016, 2018a,b). In 2016, my co-author and I reported the results of a Stroop task in which English-Spanish bilinguals, English-Other bilinguals (a group that included a wide range of language backgrounds), and English monolinguals responded to Spanish and English color words (Incera and McLennan, 2016). We found that initiation times (the time it takes to start moving the mouse) were longer for the English-Spanish bilinguals, followed by the English-Other, and the English monolinguals. However, the overall trajectory was more efficient (straighter/faster) for those who took longer to start moving the mouse. In light of these results, we argued that bilinguals are qualitatively (as opposed to quantitatively) different from monolinguals. We proposed that this pattern of results indicates that bilinguals are experts at managing information (Incera and McLennan, 2016).

Results from our study provided initial support for the *Bilingual Expertise Hypothesis*, the idea that bilinguals are experts at managing information. The expertise pattern (i.e., longer initiation times coupled with more efficient responses) has been recently replicated in a study in which English monolinguals and Chinese-English bilinguals were compared using the Flanker, Simon, and Spatial Stroop tasks (Damian et al., 2018). Furthermore, this pattern also emerged in a Master’s Thesis about attentional switching that compared bilingually exposed infants to their monolingual counterparts (Kakvan, 2017). Just as experts in a variety of domains (e.g., baseball) have a slower initiation of response followed by more efficient performance (Shank and Haywood, 1987; Incera and McLennan, 2016), bilinguals across different tasks show this expertise pattern.

The *Bilingual Expertise Hypothesis* can also be connected to the literature regarding the long term consequences of language experience. According to the *Adaptive Control Hypothesis* (Green and Abutalebi, 2013), language control processes adapt to the recurrent demands placed on them by the interactional context. One of the ways in which this adaptation might occur is that bilinguals become experts at managing their languages. If that is the case, changes due to language exposure will not simply result in participants becoming “faster” or “slower” at responding to a particular task. Instead, language exposure could qualitatively alter the unfolding of participants’ responses. Furthermore, changes across the lifespan that influence cognitive processes could also interact with the expertise pattern. For example, older adults might take longer to initiate mouse movements regardless of their language background, an aging pattern that could obscure expertise effects in older groups. The short and long term consequences of bilingualism are likely to interact, resulting in a variety of patterns that researchers need to disentangle.

It is important to acknowledge that the expertise pattern not always emerges when comparing bilinguals and monolinguals in a mouse-tracking task. In a recent study, my co-author and I used a similar Stroop task to investigate bilingualism across the lifespan and did not find differences in initiation times (Incera and McLennan, 2018a). There are several differences between our 2016 and our 2018 study that could explain these apparently contradictory findings. First, in the 2016 study we presented four response alternatives in the screen (RED YELLOW – BLUE GREEN), while in the 2018 study there were only two (RED – GREEN). The working memory capacity necessary to keep in mind four (as opposed to two) responses could have enhanced the expertise pattern. Second, in the 2016 study Spanish and English words were presented randomly, while in the 2018 only English words were presented. Being in bilingual mode might be more likely to result in the emergence of the expertise pattern, a possibility supported by the fact that in the original experiment the expertise pattern was more pronounced in the English-Spanish bilinguals than the English-Other bilinguals. These results point to the idea that task characteristics are likely to influence the unfolding of participants’ responses.

Another interesting aspect of the Incera and McLennan’s (2018a) study is that, contrary to previous research (Bialystok et al., 2004, 2008; Blumenfeld et al., 2016), no Bilingualism by Age interaction emerged. Instead, our results suggest that after controlling for baseline performance the bilingual advantage remains stable across the lifespan. Consequently, it is important to control for baseline motor differences between groups. Choices like the distance or size of the target can alter the mouse trajectory (Walker et al., 1997). Controlling for differences in motor movements is particularly important in quasi-experimental approaches–when comparing participants that cannot be randomly assigned to groups. To evaluate the influence of personal variables (e.g., bilingualism, age), it is necessary to distinguish effects at the motor level from those arising at the cognitive level. To do so, I strongly encourage researchers to add a baseline measure to their studies (see Incera and McLennan, 2018a, for an example of a baseline task).

Another important consideration to be mindful of when analyzing mouse-tracking data is the abundance of dependent variables. MouseTracker (Freeman and Ambady, 2010) provides numerous overall variables that summarize the trajectory using a single number: initiation time, reaction time, maximum deviation, area under the curve, maximum deviation time, *x*-flips, and *y*-flips. Based on preliminary analyses of the data collected in my lab, most of these variables tend to load onto two factors: (1) how straight are the mouse movements? (area under the curve, maximum deviation, *x* flips) and (2) how fast are the mouse movements? (initiation time, reaction time, maximum deviation time). Additional factor analyses are necessary to properly evaluate whether these two factors remain stable across different populations and tasks. Moreover, factor analysis is a powerful methodology to summarize across a wide range of independent variables traditionally used in bilingual research (Marian et al., 2007; Anderson et al., 2018a,b).

The key advantage of mouse tracking is that this paradigm provides measures that unfold over time: *x*-coordinates, *y*-coordinates, velocity, acceleration, and angle. The most commonly reported dependent variable–and closest equivalent to proportion of fixations–is *x*-coordinates over time. When looking at the mouse trajectories (Incera and McLennan, 2018a, Figure 2), it is possible to observe that the difference in *x*-coordinates (separation of the lines) between bilinguals and monolinguals emerges around 500 ms after stimulus onset. These results follow those of Bialystok et al. (2006) eye-tracking study in that the bilingual advantage may be evident only later on in the response. If we want to represent the mouse trajectories in line with the eye-tracking figures, we should put time on the *x*-axis, and *x*-coordinates on the *y*-axis. Alternatively, it is possible to represent these trajectories to closely mimic the visual display of the actual experiment. To mimic the visual display, we need to flip the figure by putting time on the *y*-axis and the dependent variable (*x*-coordinates) on the *x*-axis. The latter approach (time: *y*-axis) is more visually appealing, but the former (time: *x*-axis) might be better aligned with the way data from eye-tracking and event-related potentials are often represented.

## Event-Related Potentials

Event-related brain potentials provide detailed information about timing (see Moreno et al., 2008, for an overview of ERPs in the study of bilingual language processing). Several research teams have investigated bilingual populations using ERPs (Liu and Perfetti, 2003; Moreno and Kutas, 2005; Ojima et al., 2005; Kotz, 2009; Van Heuven and Dijkstra, 2010; Garcia-Sierra et al., 2011; Martin et al., 2013; Grundy et al., 2017; Zirnstein et al., 2018). Researchers have used this methodology to specifically test the bilingual advantage by measuring the effects of learning a second language on brain activation (Sullivan et al., 2014; Moreno and Lee, 2015) and by comparing bilinguals’ and monolinguals’ levels of executive control (Kousaie and Phillips, 2012, 2016; Kuipers and Thierry, 2013; Coderre and Van Heuven, 2014; Moreno et al., 2014; Heidlmayr et al., 2015; Grundy et al., 2017; Zirnstein et al., 2018). In this review, I focus on studies that used the Stroop task to investigate how the cognitive processes underlying the bilingual advantage unfold over time (Kousaie and Phillips, 2012, 2016; Coderre and Van Heuven, 2014; Heidlmayr et al., 2015).

In the Stroop task (Stroop, 1935) participants need to avoid reading the word and instead report the color of the stimuli in front of them (e.g., answering “green” to the stimuli BLUE written in green font). The Stroop effect refers to the difference between the incongruent (BLUE in green) and the congruent (BLUE in blue) conditions. The Stroop task has been used in numerous studies to investigate the timing of conflict resolution (Liotti et al., 2000; Badzakova-Trajkov et al., 2009). In monolingual participants, researchers have found an effect between 400 and 450 ms (Liotti et al., 2000) or between 370 and 480 ms (Badzakova-Trajkov et al., 2009); this negative interference effect has been associated with the N400. According to Badzakova-Trajkov et al. (2009), in the Stroop task the N400 emerges in the anterior cingulate region, and it is likely to reflect the identification and resolution of conflict between reading the word and naming the color. The N400 is also an important ERP component in the bilingual literature (Kerkhofs et al., 2006; Midgley et al., 2009).

Heidlmayr et al. (2015) compared French-German bilinguals to French monolinguals in an adapted version of the Stroop task. In addition to congruent, incongruent, and control conditions participants had to respond to a negative priming condition (the color inhibited in the previous trial becomes the target color in the new trial). In line with eye-tracking and mouse-tracking studies that speculated that the bilingual advantage might only become evident relatively late during processing (Bialystok et al., 2006; Incera and McLennan, 2018a), Heidlmayr et al. (2015) found reduced ERP effects in bilinguals’ responses to the Stroop task in the N400 and in late time windows (540–700 ms). These researchers found a bilingual advantage in the N400 Stroop effect over the posterior scalp, associated with the anterior cingulate cortex. Heidlmayr et al. (2015) did not find group differences in early components (e.g., N200, P300), but the N400 Stroop effect was reduced in bilinguals when compared to monolinguals.

Kousaie and Phillips (2012, 2016) used ERPs to compare high proficient English-French bilinguals to English monolinguals in the Stroop, Simon, and Flanker tasks (Kousaie and Phillips, 2012, 2016). In the Stroop task, the P300 peaked earlier for young bilinguals than young monolinguals (Kousaie and Phillips, 2012) and the N200 peaked earlier for old bilinguals than old monolinguals (Kousaie and Phillips, 2016). It is important to highlight that Kousaie and Phillips defined the N200 between 220 and 360 ms, and the P300 between 300 and 500 ms (which technically includes the N400). When looking at the waveforms of their Stroop task (Kousaie and Phillips, 2012, Figure 2), it becomes obvious that the bilingual and monolingual lines diverge during both the P300 and the N400. In order to better compare the time-course of the bilingual advantage across studies, researchers need to report the specific time period during which bilingual and monolingual groups differ.

Coderre and Van Heuven (2014) used ERPs to compare a group of Chinese-English bilinguals to a group of English monolinguals in a version of the Stroop task in which stimulus onset asynchronies (SOAs) were manipulated (the word and the color were not always presented at the same time). Coderre and Van Heuven (2014) found a significant negative effect at Cz and Pz between 350 and 550 ms in the monolingual group and the bilingual group when tested in their native language. However, when bilinguals were tested in their second language the effect was delayed (see Mercier et al., 2014, for equivalent findings in eye tracking). It is important to highlight that the time window reported by Coderre and Van Heuven (2014) (350 – 550 ms) incorporates the previously discussed P300 (Kousaie and Phillips, 2012) and N400 (Heidlmayr et al., 2015) components.

In order to compare across studies it is important to better determine how many milliseconds after stimulus onset a particular process is expected to emerge. A helpful approach to avoid large time-windows it to report peak latencies. For example, Coderre and Van Heuven (2014) reported that the bilingual L2 incongruent effect (529 ms) peaked later than the bilingual L1 (459 ms) and the monolingual (434 ms) incongruent effect. In order to report peak latencies, the ERP averages need to be time-locked to the moment in time in which the stimulus was presented. Researchers need to carefully consider the theoretical implications of reporting stimulus-locked (time-locked to the moment in time in which the stimulus was presented) or response-locked (time-locked to the response of the participant) ERP averages. In order to compare ERP responses to eye-tracking and mouse-tracking responses, I recommend reporting responses locked to the moment in time in which the stimulus was presented.

Crucially, Coderre and Van Heuven (2014) reported that in the -400 ms SOA, the bilingual L1 experienced a significantly later Stroop effect compared to monolinguals. This delayed onset of conflict processing in bilinguals could be indicative of enhanced inhibitory control. Coderre and Van Heuven (2014) discuss these findings in line with the dual control theory (De Pisapia and Braver, 2006; Braver et al., 2009). According to Braver and Colleagues (2009), there are “two mechanisms of cognitive control: one a “late correction” reactive response engaged to resolve conflict once it has occurred; and one a proactive “early selection” strategy engaged to pre-emptively reduce control demands for when conflict occurs.” (Coderre and Van Heuven, 2014, p. 13). This dual control theory aligns with the predictions derived from the *Bilingual Expertise Hypothesis*. First, the proactive “early selection” strategy could be the reason why bilinguals take longer to start moving the mouse. Second, the “late correction” reactive response relates to how bilinguals respond faster later on. Differences between bilinguals and monolinguals could emerge from alternative ways of processing information through these two mechanisms of cognitive control.

## Integration

Triangulating eye-tracking, mouse-tracking, and ERP measures can be tremendously useful in painting a clearer picture of the timing of the bilingual advantage. When trying to evaluate the timing of a particular task across different techniques it becomes obvious that there are numerous gaps in the literature. However, the few studies that have focused on timing point to the conclusion that investigating the unfolding of participants’ responses can help improve our understanding of the differences between bilinguals and monolinguals. In order to move forward it is important to (1) use the same sample and task across different techniques, (2) use the same task and technique across different samples, and (3) use the same technique and sample across different tasks. The type of task being used, and the cognitive processes engaged in that particular task, are likely to influence the timing of participants’ responses. Only by triangulating across samples, tasks, and techniques it will be possible to understand the timing of the cognitive processes driving these effects.

Pioneer researchers have already made efforts to integrate eye tracking and mouse tracking in their work with bilinguals. Bartolotti and Marian (2012) reported eye-tracking and mouse-tracking data collected within the same task. These researchers trained bilingual and monolingual participants in an artificial language to be able to compare them. Participants listened to spoken words and had to choose from pairs of drawings in the screen (Bartolotti and Marian, 2012). According to their eye-tracking data, bilingual and monolingual participants experienced similar early activation of the native-language competitor (200 ms after word onset) but bilinguals resolved the competition faster than monolinguals (700 ms vs. 1400 ms). While Bartolotti and Marian (2012) used the mouse-tracking results to discuss how bilinguals and monolinguals differ in the way in which they manage competition, they did not report specific timing information derived from the mouse trajectories.

Bartolotti and Marian (2012) reported the normalized, as opposed to the raw, mouse trajectories (this distinction relates to the previously mentioned way of plotting ERP data by using stimulus-locked vs. response-locked averages). The normalized mouse trajectories standardize participants’ responses by dividing each trajectory in 100 bins. These bins include longer time windows for slower participants (e.g., 50 ms per bin for someone who took 5000 ms to respond) and shorter time windows for faster participants (e.g., 10 ms per bin for someone who took 1000 ms to respond). Normalized trajectories can be useful to answer questions like: what was the position of the mouse half way through the response? However, raw mouse trajectories are necessary to answer questions like: how many milliseconds after stimulus onset does the bilingual advantage emerge? Researchers can only examine the average time at which a particular effect emerges using raw trajectories (e.g., *x*-coordinates over time).

In addition to measuring participants’ responses to the same task using different techniques, it is important to analyze the data in an integrated way. Researchers tend to report results from different dependent variables in separate sections. I recommend creating a paragraph within the results section in which the outcomes from different techniques can be integrated (similar to the “General Discussion” when reporting several experiments). It would be helpful to plot the eye- and mouse-tracking data in a single plot, and to discuss the similarities and differences of the timing across these techniques. Importantly, the way in which the data from these different methodologies converge can be as informative as the way in which they differ.

## Suggestions

Combining time-sensitive techniques can be extremely useful when trying to understand the time course of the cognitive processes underlying executive function. However, it is important to keep in mind that using different methodologies can pose technical challenges and increase the complexity of the statistical analyses. Team collaborations, in which different researchers are experts in a variety methodologies, can be highly effective in overcoming these difficulties. Furthermore, it is important to pre-register specific hypotheses for each technique, in particular when differences between these methodologies are likely to emerge. Triangulating across techniques can substantially increase the number of dependent variables, so researchers need to clearly distinguish between confirmatory and exploratory analyses.

Numerous analytical innovations have been proposed in an effort to shed new light on the discussion surrounding the bilingual advantage (Woumans and Duyck, 2015; Calvo et al., 2016). Useful methodological advances like multiverse analysis–performing all analyses across the whole set of alternatively processed data sets corresponding to a large set of reasonable scenarios (Steegen et al., 2016)–are being implemented to investigate whether arbitrary analytical choices can influence the effects of language usage on executive function (Poarch et al., 2018). Since it is virtually impossible to perfectly match bilinguals and monolinguals (Filippi et al., 2018), it is important to control for baseline levels of performance and to focus on the group by condition interactions–as opposed to the main effect of bilingualism (Incera and McLennan, 2018a). In addition, including trial presentation order as a control variable (Mercier et al., 2014) can help eliminate noise and improve the quality of the analysis.

When using statistical analyses to investigate responses over time it is crucial to properly model the covariance structure. When data points are collected over time, it is logical to assume that measures of the same participant are correlated. Data points that are closer together tend to correlate more than data points that are farther apart, which challenges the assumption of random error. Therefore, time analyses must address the issue of covariation between time points. Ignoring the covariance structure when modeling time can lead to erroneous inferences (Littell et al., 2000; Lui et al., 2012). According to Littell et al. (2000) the choice of the covariance structure can have important effects on tests and estimates of fixed effects. Lui et al. (2012) argued that researchers need to empirically consider what type of error structure best fits the data. To do so, they recommend using AIC and BIC in the selection of a proper residual covariance structure. The Akaike information criterion (AIC) and the Bayesian information criterion (BIC) are tools to compare statistical models in order to choose the best fit for a given set of data. Covariation can be a problem when analyzing timing data, researchers need to ensure they are choosing models with the right covariance structure.

A key question that researchers focusing on timing need to consider is whether “time” should be treated as a categorical (Kousaie and Phillips, 2012, 2016; Mercier et al., 2014) or as a continuous (Blumenfeld et al., 2016; Incera and McLennan, 2016, 2018a) variable (for a discussion of the statistical implications of this choice see Lui et al., 2012). The advantage of treating time as a categorical variable is that you can use specific time windows (e.g., P300, N400) to compare across studies. In addition, this approach simplifies the statistical analyses and allows for clearer *a priori* predictions. However, focusing on 100 ms time bins is a crude approach when the goal is to better understand the timing of the effects. Researchers have argued against the practice of categorizing continuous variables (MacCallum et al., 2002) and in favor of treating time (and bilingualism) as continuous variables (Incera and McLennan, 2018a). Approaches like growth curve analysis (Mirman, 2016), latent growth curve analysis (Ferrer et al., 2008), and piecemeal growth curve analysis (Calet et al., 2015), can be useful when treating time as a continuous variable. These methodologies take into account the overall pattern of the trajectory instead of focusing on arbitrary time windows.

Temporal differences are often easy to visualize in figures, but relatively difficult to pinpoint with our current statistical methods. For example, in a mouse-tracking study in which a group of Spanish-English bilinguals participated in a Stroop task with Spanish and English color words (Incera and McLennan, 2018b), my co-author and I reported that within-language interference (English words with English response alternatives) emerged 80 ms earlier than between-language interference (Spanish words with English response alternatives). It is obvious that if we had used 100 ms time-windows we would have missed this 80 ms time difference. Instead, we performed 50 within-participants *t*-tests (one every 20 ms) for the first 1,000 ms of the mouse trajectories. To maintain the overall Type-I error rate below 0.05, we used Monte Carlo simulations to calculate the minimum threshold of contiguous *t*-tests that had to be significant in order to consider the effect real (for a detailed explanation of this approach, see Dale et al., 2007; Yamamoto et al., 2016). Using this threshold, we observed that interference emerged 420 ms after stimulus onset in the within-language condition and 500 ms after stimulus onset in the between-language condition, which led us to conclude that the difference is 80 ms.

To my knowledge, there is no clear path to test whether this 80 ms temporal difference is a real effect above and beyond random chance. One approach could be to perform 50 ANOVAs, but establishing thresholds using Monte Carlo simulations would become increasingly difficult. Another approach could be to use growth curve analysis. However, it is not clear how researchers can use this technique (a tool that was created to evaluate the overall pattern of the trajectory) to pinpoint the moment at which two trajectories diverge. Even piecemeal growth curve analysis can be limited when the goal is to evaluate timing because researchers tend to use theoretical reasons (not empirical analyses) to select the time periods for the different growth patterns. As such, developing new statistical approaches that researchers can use to specify the moment at which a particular cognitive process influences participants’ responses (e.g., an analysis of the point of divergence between two trajectories or the inflection point within a single trajectory) is an important endeavor likely to inform other areas of psychological science.

## Conclusion

While data on the timing of the bilingual advantage are scarce, the empirical evidence available suggests that the effects of language experience unfold differently in the bilingual mind than in the monolingual mind. Bilinguals may be more efficient processers than monolinguals, but those effects may only be evident at certain points in time, and may differ across different samples and tasks. Understanding the timing of these effects can help explain why and how bilinguals process information differently. Therefore, it is crucial to take advantage of temporally sensitive methodologies such as eye tracking, mouse tracking, and ERPs, in order to better understand the bilingual advantage.

Investigating the timing of the bilingual advantage has the potential to stimulate new research questions and provide novel insights. Focusing only on the final outcome of participants’ responses can lead to inconclusive results because of subtle time differences in the unfolding of the underlying cognitive processes. In addition to many other important aspects of the bilingual experience (e.g., sample characteristics, task characteristics), researchers need to consider the timing of the cognitive processes at play. Methodological and analytical innovations to better investigate the timing of the bilingual advantage have the potential to inform a wide range of areas in psychological science.

## Author Contributions

The author confirms being the sole contributor of this work and approved it for publication.

## Conflict of Interest Statement

The author declares that the research was conducted in the absence of any commercial or financial relationships that could be construed as a potential conflict of interest. The reviewer EM andhandling editor declared their shared affiliation at the time of the review.
